# [Retracted] MicroRNA-30e inhibits adhesion, migration, invasion and cell cycle progression of prostate cancer cells via inhibition of the activation of the MAPK signaling pathway by downregulating CHRM3

**DOI:** 10.3892/ijo.2024.5670

**Published:** 2024-07-15

**Authors:** Xin-Min Zheng, Peng Zhang, Man-Hua Liu, Ping Chen, Wei-Bing Zhang

Int J Oncol 54: 443-454, 2019; DOI: 10.3892/ijo.2018.4647

Following the publication of the above article, a concerned reader drew to the Editor's attention that certain of the Transwell invasion assay data shown in Fig. 7B on p. 451 were strikingly similar to data that had appeared in Fig. 3D in a previously published paper written by different authors at a different research institute, which had been received at the journal *Cancer Letters* at around the same time, and which has subsequently been retracted [Gu J, Wang Y, Wang X, Zhou D, Shao C, Zhou M and He Z: Downregulation of lncRNA GAS5 confers tamoxifen resistance by activating miR-222 in breast cancer. Cancer Lett 434: 1-10, 2018]. In addition, there were potentially anomalous features associated with the western blot and cell cycle data in this paper.

In view of the fact that certain of the data in the above article were also submitted to a different journal within the space of a few days, the Editor of *International Journal of Oncology* has decided that this paper should be retracted from the publication. The authors were asked for an explanation to account for these concerns, but the Editorial Office did not receive a reply. The Editor apologizes to the readership for any inconvenience caused.

